# Conceptualizing eating disorder recovery research: Current perspectives and future research directions

**DOI:** 10.1186/s40337-022-00678-8

**Published:** 2022-11-15

**Authors:** Heather Hower, Andrea LaMarre, Rachel Bachner-Melman, Erin N. Harrop, Beth McGilley, Therese E. Kenny

**Affiliations:** 1 Department of Psychiatry, Eating Disorders Center for Treatment and Research, University of California at San Diego School of Medicine, 4510 Executive Drive, San Diego, CA 92121 USA; 2 Department of Health Services, Policy, and Practice, Hassenfeld Child Innovation Institute, Brown University School of Public Health, 121 South Main Street, Providence, RI 02903 USA; 3 School of Psychology, Massey University, North Shore, Private Bag 102-904, Auckland, 0632 New Zealand; 4 Clinical Psychology Graduate Program, Ruppin Academic Center, 4025000 Emek-Hefer, Israel; 5 School of Social Work, Hebrew University of Jerusalem, Mt. Scopus, 9190501 Jerusalem, Israel; 6 Graduate School of Social Work, University of Denver, 2148 S High Street, Denver, CO 80208 USA; 7 University of Kansas School of Medicine, 1010 N Kansas St, Wichita, KS 67214 USA; 8 Department of Psychology, Clinical Child and Adolescent Psychology, University of Guelph, 50 Stone Road East, Guelph, ON N1G 2W1 Canada

**Keywords:** Eating disorders, Recovery, Quantitative research, Qualitative research, Multi-methods research

## Abstract

**Background:**

How we research eating disorder (ED) recovery impacts what we know (perceive as fact) about it. Traditionally, research has focused more on the “what” of recovery (e.g., establishing criteria for recovery, reaching consensus definitions) than the “how” of recovery research (e.g., type of methodologies, triangulation of perspectives). In this paper we aim to provide an overview of the ED field’s current perspectives on recovery, discuss how our methodologies shape what is known about recovery, and suggest a broadening of our methodological “toolkits” in order to form a more complete picture of recovery.

**Body:**

This paper examines commonly used methodologies in research, and explores how incorporating different perspectives can add to our understanding of the recovery process. To do this, we (1) provide an overview of commonly used methodologies (quantitative, qualitative), (2) consider their benefits and limitations, (3) explore newer approaches, including mixed-methods, creative methods (e.g., Photovoice, digital storytelling), and multi-methods (e.g., quantitative, qualitative, creative methods, psycho/physiological, behavioral, laboratory, online observations), and (4) suggest that broadening our methodological “toolkits” could spur more nuanced and specific insights about ED recoveries. We propose a potential future research model that would ideally have a multi-methods design, incorporate different perspectives (e.g., expanding recruitment of diverse participants, including supportive others, in study co-creation), and a longitudinal course (e.g., capturing cognitive and emotional recovery, which often comes after physical). In this way, we hope to move the field towards different, more comprehensive, perspectives on ED recovery.

**Conclusion:**

Our current perspectives on studying ED recovery leave critical gaps in our knowledge about the process. The traditional research methodologies impact our conceptualization of recovery definitions, and in turn limit our understanding of the phenomenon. We suggest that we expand our range of methodologies, perspectives, and timeframes in research, in order to form a more complete picture of what is possible in recovery; the multiple aspects of an individual’s life that can improve, the greater number of people who can recover than previously believed, and the reaffirmation of hope that, even after decades, individuals can begin, and successfully continue, their ED recovery process.

## Plain English summary

How we research eating disorder (ED) recovery impacts what we know (perceive as fact) about it. In this paper we aim to provide an overview of the ED field’s current perspectives on recovery, discuss how our methodologies shape what is known about recovery, and suggest a broadening of our methodological “toolkits” in order to form a more complete picture of recovery. To do this, we (1) provide an overview of commonly used methodologies (quantitative, qualitative), (2) consider their benefits and limitations, (3) explore newer approaches, including mixed-methods, creative methods (e.g., Photovoice, digital storytelling), and multi-methods (e.g., quantitative, qualitative, creative methods, psycho/physiological, behavioral, laboratory, online observations), and (4) propose a potential future research model with a multi-methods design, incorporating different perspectives (e.g., increasing recruitment of diverse participants, including supportive others in study co-creation), and a longitudinal course (e.g., capturing cognitive recovery, which often comes after physical). In this way, we seek to expand our picture of what is possible in recovery; the multiple aspects of an individual’s life that can improve, the greater number of people who can recover than previously believed, and the reaffirmation of hope that, even after decades, individuals can begin and continue their ED recovery process.

## Background

How we research the process of eating disorder (ED) recovery impacts what we know (perceive as fact) about this process. Traditionally, research has focused more on the “what” of recovery (e.g., establishing criteria for recovery, connecting research and clinical experiences, reaching consensus definitions) than the “how” of recovery research (e.g., timing and framing of ED recovery items and measures, type of methodologies, triangulation of perspectives). Given that our “ways of looking” are inextricably tied to what we are looking *at* [1] it is important to step back and investigate how research methods shape what we can know about a phenomenon of interest. This exploration can offer insight into missing pieces of the analytic puzzle (e.g., the current gaps in our knowledge), and invite novel ways of researching ED recovery (e.g., incorporating different perspectives).

ED recovery research that is published in peer-reviewed journals most frequently uses quantitative (numerical, “objective”)^1^ statistical methods, or qualitative (descriptive, “subjective”) interview methods, in order to convey their findings. In this paper, we provide an overview of commonly used methods and outline key analytic features of various types of analyses that fit within these broader method categories. We also present an examination of these commonly used methods, reflecting on the benefits and limitations of each, and what each allows us to know, or not know, about ED recovery. Following this overview, we explore mixed-methods (quantitative and qualitative), creative methods (e.g., Photovoice, digital storytelling), and multi-methods (e.g., quantitative, qualitative, creative methods, psycho/physiological, behavioral, laboratory, online observations), which may provide directions for future research, and enable new understandings of ED recovery.

Importantly, we are not suggesting that researchers abandon these commonly used quantitative or qualitative methods or that one approach is inherently better than others. Rather, we are recommending a broadening of our methodological “toolkits,” to increase the clarity of purpose of our studies, along with an alignment of the methods used. This may enable the development of more nuanced and specific insights about ED recoveries that take into account context, varied perspectives, and different positionalities. In this paper we thus aim to provide an overview of the ED field’s current perspectives on recovery, illuminate how those perspectives are necessarily informed by our methodological choices, and recommend broadening our methodological “toolkits” in order to form a more complete picture about what can be known as possible in recovery.

## Main text

### Ontological and epistemological stances in ED recovery research

The goal or purpose of ED recovery research depends largely on the ontological (the “what” of research) and the epistemological (the “how” of research) stances endorsed by the researcher [2, 3]. This is often considered to be foundational in qualitative research, but is less frequently named in quantitative approaches. We give a brief overview of these stances here, because how a researcher views the world will inevitably impact the goal of the research and the methodological approaches used. In this way, we cannot present a discussion of recovery methodologies without also considering ontology and epistemology.

Ontological stance refers to what we believe can be known [4, 5]. From a ***realist*** perspective, there is a single objective truth which exists [6]. On the other side, a*** relativist*** perspective suggests that there is no singular reality outside of human practices [7]. Researchers can therefore vary along this spectrum in terms of their assumptions about what knowledge exists.

Epistemology refers to how we can come to know this information [8]. For example, ***positivism*** argues that we can come to understand or know an objective reality through rigorous scientific practices [9, 10]. This is the foundation of the scientific approach and what has often been referred to as the “hard sciences.” Recently, there has been a shift in which individuals from this perspective acknowledge that data collection and interpretation may be imperfect and influenced by researcher characteristics; what is now known as ***post-positivism*** [11].

Many of the quantitative approaches that will be discussed in this paper come from a post-positivist framework in that they assume there is an objective recovery “truth” that can be uncovered if we are rigorous in our approach and seek to minimize bias. On the other side, there are ***contextualist*** [12] and ***constructionist*** [13] epistemologies. Contextualism situates knowledge and the people who create it (e.g., participants, researchers) in a broader context, acknowledging that no one person can know everything. Constructionism argues that meaning is multiple, socially-constructed, and connected to wider systems of power. In this way, there is no one definition or understanding of a phenomenon.

The ontological/epistemological stance and research assumptions that dictate the approaches we take in turn inform debates on recovery. Those coming from different traditions will thus have different views of what can be known about the phenomenon. For example, the frames of (post)positivism typically underlie quantitative research, and researchers coming from this perspective have long been calling for a clear, consistent, and applicable definition of recovery (e.g., [14–17]). However, no overall consensus definition has been reached to date, which has several implications from a (post)positivist perspective. This lack of conceptual clarity, and between-study differences in measurement approaches, impact our ability to compare the findings between studies, including reported recovery rates, which can vary dramatically, depending upon the definitions and clinical groups used (e.g., [14, 15, 18, 19]).

The belief that there is a need for a singular definition is one way of understanding the utility of recovery and may be useful for some groups. The intent of our paper, though, is not to provide a statement about what a consensus definition might be. Rather, we are offering a more diverse view of methodological perspectives (which stem from various ontological and epistemological stances) and ideas that might allow for forward movement in the field. In a dialectal format, this can involve both movement toward *and* away from consensus, including perspectives which do not seek to identify a single recovery definition. These paths are sometimes polarized, indicating that research aiming for (provisional) consensus is incompatible with research pushing into new areas. We suggest that both can be simultaneously pursued, acknowledging that one does not discount the other.

### ED recovery research approaches: A brief overview

Quantitative research stemming from (post)positivist perspectives has tended to emphasize “objective” illness and recovery criteria that can be measured and compared in the lab/treatment, such as body mass index (BMI) (e.g., [20]), and behavioral/cognitive symptoms (e.g., [21, 22]). For example, scores of validated ED measures such as the Eating Attitudes Test (EAT) [23] are frequently subdivided into “threshold” (criteria met for a probable clinical diagnosis) or “subthreshold” (diagnostic criteria unlikely to be met). Changes in measurable physical, behavioral, and symptomatic criteria are characteristic of the medical model of recovery, with a growing body of research suggesting that such approaches may not fit as well with lived experience perspectives [24, 25].

In a systematic review of 126 studies looking at predictors of ED outcomes [26], symptom remission was used as a key outcome in over 80% of studies. This may differ from the “process” recovery criteria typically used in clinical settings, where the individual’s progress in therapy (e.g., how they navigate their recovery, showing improvements in not only symptoms, but also psychosocial functioning) may affect the extent to which they are deemed “recovered.”

A key element of these different definitions hinges on the extent to which symptom remission is considered an important first step in recovery. This point has often been promoted as self-evident, but is inconsistent with some orientations to recovery. For instance, a recovery model orientation, which has been noted to be potentially resonant with EDs (e.g., [27]) starts with an emphasis on a person’s goals and contexts, rather than assuming that symptom remission is a first step. This does not mean that “anything goes"; a recovery model promotes collaboration and discussion in exploring what recovery means and does for the person seeking it [28, 29].

More recently, researchers have suggested that in alignment with this recovery model, it may be possible to continue to exhibit some symptoms (e.g., behaviors), but have improvement in other areas (e.g., improved psychosocial functioning, QOL), and still feel that one is in ED recovery (e.g., [27, 29–35]). ED advocacy groups led by people with lived experience are also beginning to support approaches to harm reduction within ED recovery circles, such as those promoted by Nalgona Positivity Pride. Indeed, there are now several ED-specific, standardized measures of functioning and QOL that can provide more insight in this area, including the Eating Disorders Quality of Life (EDQOL) [36], Quality of Life for Eating Disorders (QOL ED) [37], Health-Related Quality of Life in Eating Disorders Questionnaire (HeRQoLED) [38], Eating Disorders Quality of Life Scale (EDQLS) [39], and, more recently, the Eating Disorders Recovery Questionnaire (EDRQ) [40]. In addition to assessing QOL from a quantitative perspective, QOL can also be explored qualitatively, allowing it to be contextualized against the landscape of participants’ lives.

What is considered to “matter” in ED recovery definitions thus far also differs to some extent according to who is asked. Researchers, clinicians, and people with lived experience (individuals and supportive others such as parents, family, partners, friends, mentors) may emphasize different criteria for ED recovery [41]. Further, these categories are not distinct; people may simultaneously occupy multiple positionalities at once, such as researchers and/or clinicians who also have lived experience of ED. While a consensus definition among clinicians is arguably becoming a more plausible goal [16], there may still be significant divergence of opinion between the larger clinical, research, and lived experience spaces [18]. Nevertheless, Bachner-Melman et al. [42] also found a broad area of overlap in the perspectives of people with lived experience of an ED, family members, and ED therapists, on what recovery encompasses. They proposed a questionnaire to measure four aspects of ED recovery that were agreed on by these overlapping perspectives: lack of symptoms, acceptance of self and body, social and emotional connection, and physical health [20].

Lived experience, including personally having lived with an ED, as well as being a “support” for someone with an ED (e.g., parents, family, partners, friends, mentors), necessarily informs a particular person’s ED recovery definition and provides an additional lens on the same construct. Thus, individuals who have lived through an ED may have a different view of recovery to that of their “supports” (e.g., improved psychosocial functioning, QOL, vs. medical stability, decreases in behaviors, and vice versa). However, the recovery priorities of individuals and “supports” may also align. For example, recent studies have indicated that both individuals and parents/families place high value on increased body acceptance and independence in the individual’s recovery process [43, 44]. In addition, there is a relatively new resource for partners of those with ED, which focuses on understanding, supporting, and connecting with the partner on shared recovery goals [45]. In the book “Loving Someone with an Eating Disorder,” Dana Harron includes perspective-taking exercises to help the person understand their partner’s struggle, strategies for dealing with mealtime challenges, up-to-date facts about EDs, and self-care tips to help the person maintain healthy boundaries [45].

### Conceptual and methodological challenges in ED recovery research

Related to the above, researchers face a number of conceptual and methodological questions when exploring ED recovery. For example, whether or not recovery should be considered per individual ED, or trans-diagnostically, is an important question in the recovery definition literature. Bardone-Cone et al. [15] suggest that a transdiagnostic approach is most appropriate, given that diagnoses can shift, and symptoms can fluctuate over time. Indeed, longitudinal studies have indicated that participants report receiving a single ED diagnosis at one point in time, however, over their lifetime, they would have met criteria for two, three, or four “different” ED diagnoses at different times (e.g., [46]). Anecdotally, our co-authors have also noted this when recruiting participants for research. Instead of necessitating an overall cessation of ED symptoms within one diagnostic category in the traditional categorical approach, a transdiagnostic approach could rather focus on improving the status of individual symptoms (e.g., frequency of restricting, binging) as a marker of individual “recovery.”

Beyond differences in being able to compare clinical groups across research findings, we might also consider who is most commonly included in these recovery studies (and who is not). There are many significant logistical barriers to receiving an ED diagnosis and related treatment worldwide. Indeed, practical barriers include: cost, insurance coverage, rurality, transportation, work or education schedules, and lack of available childcare, which disproportionately affects people from potentially disadvantaged groups (e.g., [47–50]). The process of recovery itself invokes privilege (e.g., who is able to be diagnosed, who has access to formal treatment, and who is recovering in the “right way”). For example, EDs may be missed, or diagnoses delayed, in those who do not fit the stereotypical picture of a person with an ED, including those in larger, or non-emaciated, bodies [51, 52].

The majority of the studies thus far on ED recovery definitions are therefore composed predominantly of non-diverse participant samples who have the means to overcome the barriers to treatment access (i.e., predominantly White, thin, socioeconomically privileged, cisgender women, drawn primarily from clinical settings). Indeed, there has been comparatively little research on other populations with EDs (e.g., cis men, trans and nonbinary people, children, elders, higher weight individuals, individuals with binge eating disorder [BED], comorbidities, or late onset), as these groups often do not have access to the diagnoses and treatments that are the gateway to research study participation. These limitations determine whose recoveries we can learn about, and excludes other experiences [18, 53, 54].

Traditionally, those with lived experience have not been invited to co-design recovery research, limiting study participation and the diversity of representation. Even when recovery research includes non-clinical samples, methodology choices impact who is selected for participation. For example, studies that exclude potential participants with BMIs above certain levels (e.g., BMIs that are considered “overweight” or “obese”) exclude many ED recovery experiences automatically, limiting the view of what “recovery” looks like.

Additionally, the specific terminology of “recovery” may not resonate with all people experiencing life beyond an ED [55], causing some potential participants to self-select out of such studies. Some people with lived experience note that the term “recovery” is prescribed and carries preconceptions [56, 57]. Indeed, there are nuances and connotations involved with the use of the word “recovery.” Some individuals may consider themselves “in recovery” (on a continuous journey), while others may consider themselves “recovered” (having moved past the ED completely). In this way, the meaning of “recovery” can indicate both a process and a state [58]. Stringent criteria for including people in studies as “recovered” may pre-define the group with whom recovery is being explored. Other terminology, such as severe and enduring anorexia nervosa (SEAN), and severe and enduring eating disorders (SEED), emphasize more chronic conditions. However, these terms are not always helpful for people experiencing longer-lasting ED, as they may insinuate that healthcare providers (or the patient) have given up hope for recovery [59–62].

Given that “recovery” as a term does not resonate with all [55, 63–65], using other terms, including non-clinical ones (e.g., “getting better”, “healing”) to refer to these experiences may increase the diversity of experiences in the literature. As we will explain, these methodological features matter in recovery research because they significantly impact what we can know about ED recovery, and for whom.

### Positioning ourselves

We come into this work from various vantage points; we name our positionalities here, since researchers’ subjectivity inevitably shapes their research and interpretations [66]. Engaging with the subjective, rather than presuming objectivity is the most ethical and effective stance in research, and can invite opportunities to uncover new and different knowledge [67]. The authors bring research and clinical lenses to bear on this work; some of us are primarily or exclusively researchers in the ED field, whereas others also practice clinically. We come from Global North countries, and all of us are White. We were thus trained in scientific traditions that privilege certain ways of knowing and doing that reflect the English and White dominant landscape of academia. While most of us benefit from thin privilege, able-bodied privilege, and cis-hetero privilege, our authorship team also includes those with non-binary, queer, fat, and chronically ill identities. Some of us have lived experience with ED, and have used this to inform our research and clinical practice. Some of us are newer to the ED field, whereas others have been working in the field for over 30 years. While we are different in some ways, our sameness centers around the academic privilege we have to access, interpret and navigate these literatures and their methodologies.

### Overview and analysis of ED research methods

Below we provide an overview of the commonly used quantitative and qualitative research methods, along with tables that illustrate examples of the different types of analyses that fit within these broader methodological categories. We also analyze the benefits and limitations of each method, focusing on what we can learn from them and identifying relevant gaps in the literature.

#### Quantitative methods

As noted above, quantitative methods typically stem from a (post)positivist ontological/epistemological stance, which inherently affects how data are interpreted and understood. This is a core consideration of how we in turn can view the findings. This approach aims to provide “objective” results [68]; in this case, it is “recovery by the numbers.” It allows for the measurement of results through data, relying on a systematic approach of empirical investigation, and based on the assumption that there is a singular recovery definition which can be known. Researchers use statistical models, computational techniques, and mathematics to develop and test specific hypotheses. The types of quantitative analyses range from relatively simple descriptive/comparative measures to more complex multivariate measures and multi-level designs (which are all influenced by their study samples, assessments, and testable hypotheses). Data can be collected from the traditional in-person research study (or through video conferencing), or alternatively, from participant surveys (e.g., online, phone, mail, text).

Different types of quantitative methods have been employed in ED recovery research (see Table 1). Descriptive studies focus on the “how/what/when/where,” rather than the “why” (e.g., examining aspects of recovery definitions [69]), and comparative studies have a procedure to conclude that one variable is better than another (e.g., comparing different recovery definitions for agreement [70]). Univariate analyses examine the statistical characteristics of a single variable (e.g., dichotomous yes/no variable differences between recovery groups on a single measure [33], continuous range variable differences between recovery groups on multiple measures [71]), while bivariate analyses determine the empirical relationship between two variables (X and Y) (e.g., relationships between recovery attitudes and related variables [72]). Multivariate analyses aim to determine the best combination of all possible variables to test the study hypothesis (e.g., comparing recovery and healthy control groups across different recovery scores [73]).

According to (post)positivist stances, these quantitative methods have the anticipated or theoretical benefits of enabling researchers to reach higher sample sizes (increases generalizability), randomize participants (reduces bias), and replicate results (validates data). In practice, though, generalizability extends only to the sample that is recruited (as noted above, in most cases, thin, White women), and randomization within that sample thus does not increase the diversity of results. The relative focus on “novel” research means that replication studies are not conducted to the degree we would hope or expect.

In addition, quantitative methods limit what can be known about any particular individual. For example, numbers can tell us a person’s standardized assessment scores, but they do not include the detailed descriptions of the individual’s experiences. They also reduce recovery to a single experience which may overlook the tremendous diversity in lived experiences. Similarly, while statistical analyses can account for contextual confounding variables, they cannot tell us the broader factors which influence the delivery and the function of interventions.

**Table 1:** Quantitative methods of research

Type	Description	Examples	Related literature
Descriptive	Focuses on the how/what/when/where, rather than the why	Classification of recovery criteria; examining aspects of recovery definitions	Couturier and Lock [69]
Comparative	Procedure to conclude one variable is better than another	Surveys of recovery definitions; comparing different definitions for agreement	Ackard et al. [70]
Univariate analyses	Statistical characteristics of a single variable	Statistics include distribution, central tendency, spread	
Dichotomous variables	Yes/No variables; entered into Chi Square	Differences between recovery groups on a single measure	deVos et al. [33]
Continuous variables	Range variables; entered into t-tests and ANOVA	Severity of symptoms in recovery; differences between recovery groups on multiple measures	Cogley and Keel [71]
Bivariate analyses	Determines empirical relationship between two variables (X and Y)	Statistics include correlation coefficient (r); U statistic	
Parametric	Evenly distributed data; entered into Pearson correlations	Ratings of recovery attitudes, stigma, self-esteem; relationships between recovery attitudes and related variables	Dimitropoulos et al. [72]
Non-parametric	Non-evenly distributed data; entered into Mann–Whitney–Wilcoxin or U-test	Comparing recovery groups to healthy controls	Ackard et al. [70]
Multivariate analyses	Determines best combination of all possible variables to test study hypothesis	Types of analyses: MANOVA, regressions, factor analysis, survival analysis, GEE (categorical outcomes), HLM (continuous outcomes)	
MANOVA	Determines best combination of all categorical outcome variables	Comparing recovery and healthy control groups across different recovery scores	Bachner-Melman et al. [73]

*ANOVA*  analysis of variance, *GEE*  generalized estimating equations, *HLM* hierarchical linear models, *MANOVA*  multivariate analysis of variance

### Qualitative methods

Overall, qualitative methods offer the potential to engage deeply with phenomenon of interest, often stemming from non-positivist epistemological stances (e.g., constructionist, feminist). While qualitative methods are commonly critiqued for small sample sizes, in a qualitative paradigm, small samples allow researchers to dig into the nuances illustrated in participants’ stories, strengthening study findings. The aim of qualitative research is in-depth, contextualized analysis, rather than generalizations. Qualitative methods often, but not always, involve interacting directly with participants in the form of interviews or focus groups. However, qualitative research can also involve analyses of existing textual or image data, such as blog or social media posts, or news articles. A core feature of qualitative research is the researchers’ focus on exploring meaning in voiced or textual data, vs. using only quantitative measures.

Despite shared features, qualitative methods vary enormously in terms of data collection and analysis types. This is due in part to the differences in theoretical basis, epistemologies, ontologies, and paradigms that inform what meaning researchers perceive as *possible* to achieve. Some (e.g., [74]) draw on the concepts of “big Q” and “small q” to differentiate in broad terms between the qualitative methods [75]. Briefly, “big Q” methods invite and acknowledge researcher subjectivity, whereas “small q” approaches attempt to aim at more “objective interpretation,” [76], which is more similar to (post)positivist approaches. Further, “big Q” approaches tend to delve into the connections between knowledge production, analysis, and sociocultural contexts in which research takes place, whereas “small q” approaches tend to focus more on descriptive, groundwork-laying analysis for quantitative methods to provide generalizability [76]. Neither approach is inherently “better;” they are designed to achieve distinct findings.

Some of the qualitative methods that have been commonly used to explore ED recovery experiences are summarized in Table 2. Note that these are not the *only* methods used. Some (particularly earlier) studies, describe their methods as “qualitative,” without specifying the exact type(s) of analysis. The differences between these various types of methods are at times subtle.

Discourse Analysis (DA) focuses on language not as just a route to content, but as powerful in and of itself (e.g., analysis of talk about recovery) [77, 78]. Within DA, Linguistic Analysis adds a focus on language present in the text, with more of an emphasis on terms used, and their connotations (e.g., explorations of Internet message board communications about recovery) [79]. Also within DA, Narrative-Discursive Analysis adds a focus on social power (e.g., analysis of recovery interviews with a gender lens [80]), alongside an emphasis on stories (individual and broader, social stories). Narrative Approaches emphasize the story (e.g., analyses of participant writing, life-history), and situates recovery within the broader culture [31, 81–84]). Phenomenological and Phenomenographic Approaches, including Interpretive Phenomenological Analysis (IPA) aim to get in “close” to participant embodied experiences (e.g., focus on recovery self-process in specific groups such as men, former patients, people in recovery from AN specifically [85–87]). Grounded Theory emphasizes context-specific “ground-up” theory developed from participant responses (e.g., development of cyclical, phase, and process models of recovery in/outside of treatment contexts [65, 88–91]). Thematic Analysis (TA) is aimed at developing patterns/themes based on data (e.g., exploring patterns in experiences of recovery [92–94]) and can look quite different depending on the type of thematic analysis employed, ranging from more descriptive to more analytical. Content Analysis summarizes and organizes experiences amongst a particular group in a particular context (e.g., describing the content of interviews with specific groups, for example, athletes, or exploring the content of a particular stage of recovery, such as late-stage recovery [95–99]).

It is also possible to use different qualitative methods to explore similar phenomenon. For example, a constructivist grounded theory exploration of people who have received treatment for AN may focus on *theorizing what ED recovery processes are occurring* for this particular group [99, 100]. An IPA of this same group, meanwhile, may emphasize the development of a set of themes relating to shared perspectives on what the experience *felt like* [101, 102].

Qualitative methods can thus provide detailed descriptions of a wide diversity of lived experiences. This enables us to have a broader perspective of what is possible in recovery. Additionally, these methods allow us to consider the contextual factors which influence the delivery and the function of interventions. A potential further benefit is the individual’s own process of reflecting on their changes in recovery (via study participation), which may provide insight and encouragement for continuing on their path.

Critiques of qualitative methods tend to center around the concept of generalizability, though as noted this is not typically the goal of qualitative approaches. As noted above, quantitative studies, which typically focus on a person’s ED standardized assessment scores, and account for contextual confounding variables through statistical analyses, theoretically generate findings which can be applied to other populations from the study sample. However, as we have indicated, extrapolation of the results of mostly homogenous groups (e.g., predominantly White, thin, socioeconomically privileged, cisgender women, drawn primarily from clinical settings) falsely assumes that the course and outcomes will be the same for all.

**Table 2:** Qualitative methods of research

Type	Description	Examples	Related Literature
Discourse analysis (DA)	Focus is on language as more than just a conduit of meaning, but as powerful in and of itself; explorations of how people and things are “constructed” or “formulated”, positioned, and discussed, or understood.	Analysis of talk about recovery.	Hardin [77], Malson et al. [79]
Linguistic analysis	Focus is on language and its power; adds an emphasis on language present in the text, with more of an emphasis on terms used, and their connotations.	Explorations of Internet message board communications about recovery.	Keski-Rhakonen and Tozzi [78]
Narrative-discursive	Focus is on language and its power; adds a focus on social and individual stories and how these position people and constrain/open action possibilities.	Analysis of interviews about recovery using a gender lens, focusing on how gendered power is (re)enforced through social narratives and discourses and what this opens/closes for recovery.	Moulding [80]
Narrative approaches	Emphasis is on story; either thematically (patterns of meaning related to content) or structurally (patterns of pieces of constructing stories) analyzing stories.	Interview studies exploring recovery journeys, for instance in relation to broader cultural messages about recovery and health; Analysis of participant writing about recovery; Life-history interviews and analysis of these interviews.	Dawson et al. [27], LaMarre and Rice [81], Matusek and Knudson [82], Patching and Lawler [83], Redenbach and Lawler [84]
Phenomenological and phenomenographic approaches	Getting in close to embodied experiences, linking these to perceptions and experiences of the world, including Interpretative Phenomenological Analysis (IPA).	Focusing on self-process in the experience of recovery for specific groups (e.g. men, former patients, people in recovery from Anorexia Nervosa [AN] specifically).	Björk et al. [85], Björk and Ahlström [86], Jenkins and Ogden [87]
Grounded theory	Developing localized theory that is context-specific and “ground-up” – that is, developed on the basis of participant responses	Development of cyclical, phase, process models of recovery in or outside of treatment contexts.	D’Abundo [88], Krentz et al. [89], Lamoureux and Bottorff [90], Musolino et al. [65], Woods [91]
Thematic analysis (TA)	Generally aimed at developing patterns based on data; variations within approaches to thematic analysis including codebook TA, reflexive TA, etc. These approaches have distinct approaches to the development of themes and understand themes differently.	Exploring patterns in representations or experiences of recovery, grounded in a number of different theoretical orientations that relate to how the person and their experiences relate to the world around them.	Hay and Cho [92], LaMarre and Rice [93], Lord et al. [94]
Content analysis	Describing and/or summarizing experiences amongst a particular group and/or representations in a particular context.	Describing the content of interview data related to specific groups’ (e.g. athletes, people with BN, etc.) experiences of recovery; exploring the content of a particular stage of recovery (e.g. late-stage recovery).	Arthur-Cameselle and Quatromoni [95], Lindgren et al. [96], Nilsson and Hägglöf [97], Pettersen et al. [98]

AN = Anorexia Nervosa; BN = Bulimia Nervosa; DA = Discourse Analysis; IPA = Interpretive Phenomenological Analysis; TA = Thematic Analysis

#### Exploration of mixed-methods, creative methods, and multi-methods research

While ED recovery researchers have primarily conducted either quantitative *or* qualitative studies, some have integrated alternative or multiple methods in their designs. Below we explore some of these methods, which may enable new understandings of ED recovery. These include mixed-methods (usually a weaving of quantitative and qualitative), creative methods (e.g., Photovoice, digital storytelling), and multi-methods (e.g., complementary combinations of quantitative, qualitative, creative methods, psycho/physiological, behavioral, laboratory, online observations).

#### Mixed-methods (weaving of quantitative and qualitative)

It has been argued that mixed-methods allow us to weave together our quantitative and qualitative insights by acknowledging the benefits and limits of both designs. Cluster analysis intends to combine these methods with the goal of maximizing benefits [103]. For the qualitative aspects of the analysis, data is coded to themes using one of the qualitative methods, and each individual unit of data (e.g., person) is coded for the presence or absence of each theme. For the quantitative aspects of the analysis, data is plotted to identify different clusters of individuals, and then these clusters are interpreted via statistical methods. Cluster analysis aims to provide greater insight into groups of individuals, and potentially elucidate different clusters of “recovery definitions.” However, the qualitative analysis in this approach is inherently reductionistic (e.g., people are coded to create a quantitative measure), which aligns with the post-positivist stance associated with quantitative analyses. From this view, the approaches are not actually integrated; rather they are complimentary. Indeed, it may not be possible to truly integrate them when they emerge from different epistemological stances. We suggest, however, that integration is not needed.

Bachner-Melman et al. [42] used exploratory factor analysis to identify four factors that mapped onto ED recovery which had general agreement between participants with a lifetime ED diagnosis, healthy family members, and ED clinicians; (1) lack of symptomatic behavior, (2) acceptance of self and body, (3) social and emotional connection, and (4) physical health. These factors were then confirmed using confirmatory factor analysis. Utilizing more than one method thus expands our perspectives of ED recovery, allowing us to broaden our understanding of what can be known about recovery. Yet as noted above, we caution readers in viewing mixed-method approaches as an overall panacea; the approach tends to be more (post)positivist, and aims to quantify experiences, which may not be the goal for researchers from other stances. Again, this is not to say that the approach is without merit, but that it is important to acknowledge what it aims to do (or know).

#### Creative methods (photovoice, digital/verbal storytelling, collages, drawings)

Quantitative and qualitative methods are, of course, not the only options at the disposal of researchers interested in exploring ED recovery. Some researchers have elected to take creative approaches to research, seeking to explore recovery in different ways. Potentially, such methods enable researchers to “see” facets of recovery phenomenon that are less evident in methods that primarily hinge on either words or numbers [57]. To date, ED recovery researchers have used creative methods such as Photovoice [104, 105], which aims to involve participants in the process of generating and analyzing research data [106]. This method may be particularly useful for generating disseminable results, with a view towards change in policy settings for the benefit of people in recovery [104].

Another creative method, digital storytelling [57, 107], encourages participants to “story themselves” at a particular moment in time. This may enable the creation of more nuanced, rich, and person-centered depictions of recovery; the participant’s voice is centered in a way that may be less feasible in research that seeks to generate patterns across several participants’ accounts [107]. Like Photovoice, digital stories can also be used to work toward enhancing understandings of recovery amongst people who do not have lived experience (e.g., healthcare providers) [57].

Other creative methods include the use of collages, verbal storytelling, drawing,and more [108–110]. Placing the decision about which creative method to use in the hands of research participants may also enable a redressing of traditional power dynamics in research that position the researcher as the ultimate decision-maker [57].

#### Multi-methods (complimentary use of multiple methods)

Given that ED recovery is a complex phenomenon, one approach to exploring it is the use of a multi-method research design, including different complimentary types of analyses (e.g., quantitative, qualitative, creative methods, psycho/physiological, behavioral, laboratory, online observations), and ideally from different perspectives (e.g., individuals, “supports,” clinicians, researchers, policy makers, and other stakeholders). Broadening our methodological “toolkits” may allow for more nuanced and specific insights about ED recoveries, taking into account context, varied perspectives, and positionalities.

Several studies of ED symptom assessment have employed multi-method designs thus far, and each of these has the potential to contribute a piece of the “ED recovery puzzle.” For example, Stewart et al. [111] conducted a mixed method investigation of the experiences young people, parents, and clinicians had of online ED treatment during COVID-19. They used a mixed quantitative (Likert scale rating questions) and qualitative (free text entry questions) survey which they analyzed using a summary approach (quantitative) and reflexive thematic analysis (qualitative). Leehr and colleagues [112] analyzed binge eating episodes under negative mood conditions via electroencephalography (EEG) and eye tracking (ET) in a laboratory.

Bartholome et al. [113] combined standardized instrument interviews, laboratory investigations, and ecological momentary assessment (EMA) to collect data on binge eating episodes in participants with BED. In order to examine underlying mechanisms of the somatic sensation of “feeling fat,” Mehak and Racine [114] used multiple methods of self-reports, EMA, heart rate variability, laboratory measurements of BMI, dual energy X-ray absorptiometry (DEXA) scans, and clothing sizes.

Technological advances can offer more in-depth information about the recovery process, and could be utilized further in research studies. For example, EMA allows for moment by moment collection of data on phenomenon of interest. This can include biological functions (e.g., heart rate), as well as feelings and behaviors in an individual’s daily life. This approach provides a more “real time” look into experiences (versus having to recall such experiences later on in a survey, or in an interview). In the case of ED recovery, future research can explore the answer to the question of what “active” recovery looks like on a daily basis for individuals via EMA (e.g., what challenges do individuals encounter; how does it impact their behavior/feelings?).

Other technological laboratory tools, such as fMRI, DEXA, and other scanning techniques, can add visual information about the current physical state of recovery (and any related functional “scars” from the ED). It may be helpful in future studies to provide this feedback so individuals can have an accurate picture of the medical status of their body, and make adjustments (e.g., take Vitamin D to increase bone density).

### Additional methodological approaches/considerations

#### Co-design with different perspectives in ED recovery research

We believe it will be helpful to incorporate different perspectives in ED recovery research for many reasons. For example, expanding who is included in our studies, what identities are represented, and those with both formal and informal treatment, will increase the participant representation, relevance, and generalizability of the findings [53]. This in turn will allow us to know more about the process of recovery for different individuals and groups, and will broaden our conceptualization of the phenomenon. Ideally, future research will include individuals with lived experiences and their “supports” (e.g., parents, families, partners, friends, mentors), as well as clinicians and researchers, in co-designing studies that could identify and assess aspects of ED recovery that are important to all of the constituents.

#### Longitudinal research design

We also underscore the need for an extended duration of studies in order to better understand the longitudinal course and outcome of individuals in ED recovery. This design will allow us to compare recovery operalizations vs. subsequent relapse rates, to track how perspectives of recovery develop and change over time (via quantitative, qualitative and mixed-method measures), and how these in turn affect an individual’s identity (e.g., [15]).

There are several areas of potential future longitudinal research. For example, follow-up on cognitive recovery (which we know tends to occur later (e.g., [43]), and how related timelines for this may impact subsequent relapse rates, tracking recovery changes over time with mixed or multi-methods designs in underrepresented populations (e.g., Atypical Anorexia (AAN) [115]), and holding on to hope, with more longitudinal data indicating that recovery is possible, even after decades (e.g., [59]).

### Future research directions

Based on the above studies, we suggest some potential areas for future research, ideally incorporating multi-method designs to provide different perspectives on ED recovery. Recently, several themes have been identified in the literature as promising lines of research that may improve our understanding of ED, and increase the clinical application of findings.

#### Predictors of outcomes

Within quantitative research, Bardone-Cone et al. [15] note that predictors of outcomes, biological/neuropsychological techniques, and a focus on the SEAN population are newer, more nuanced, areas of investigation. In their systematic review and meta-analysis of predictors of ED treatment outcomes (at end of treatment [EoT], and follow-up), Vall and Wade [26] reported that the most robust predictor at both time frames was greater symptom change earlier in treatment. Other baseline predictors of better outcomes included: higher BMI, fewer binge/purge behaviors, more functional relationships (e.g., with family, friends), and greater motivation to recover. Of note, it is important to understand that higher BMI is in the context of a “higher” *thin* BMI, as most people with BMI > 25 are not included in studies of recovery. This is another example of how our methods and design choices impact what we can know.

Relatedly, one potential area for future ED quantitative predictor research is to build a Risk Calculator (RC), which is a statistical tool that identifies risk factors, and determines how likely an event is to happen for a particular person [116]. Physicians have used RCs clinically across an array of medical conditions, including stroke [117] and cancer [118]. There has been a recent turn toward integrating RCs into charting psychiatric disorder outcomes and treatment approaches; they have been used for psychosis [116], depression [119], and bipolar disorder [120–123]. This same technique could be applied to build a RC for personalized risk of ED onset/relapse, utilizing variables collected in research/treatment. A statistical combination of factors that reliably predict the non-occurrence of ED relapse could be a valuable addition to, predictor of, or even criterion, for full recovery. As part of these research initiatives, it will be important for researchers to employ diverse and longitudinal methods in order to obtain long-term, dynamic data.

In line with this, narrative qualitative analysis may be useful in elucidating predictors/risk factors for individuals. In this way, the (narrative) story that the person tells themselves about their recovery, and what was helpful to them, also has importance alongside any quantitative measures. Indeed, this perspective perhaps has more personal meaning, especially in contexts where minute changes identifiable through quantitative studies may be less relevant in the daily lives of their ED recovery.

#### Biological and neurological markers

Recent developments in the understanding of biological and neurological markers have enabled us to parse out what features may be involved with the ED “state” (which resolves with recovery), what features may onset premorbid to the ED (and will potentially continue after recovery), and what features may be “scars” (consequences of the ED). In their functional Magnetic Resonance Imaging (fMRI) study on participants who had recovered from AN, Fuglset et al. [124] reported increased activation in visual processing regions in anticipation of seeing images of food, with corresponding reduced activation in decision-making regions. While they found some normalization of the brain regions during recovery, other differences related to longer periods of starvation that appear later in life remained (residual “scars”).

Future quantitative research which incorporates longitudinal designs following the same participant cohort may elucidate more closely the timepoints during which the “state” and “scar” markers begin to emerge, in order to provide earlier interventions. In this instance, qualitative longitudinal studies could be beneficial here too. For example, participant narrative descriptions of ongoing biological changes in their recovery (e.g., feeling hungrier, not being able to tolerate hunger as well) not only reaffirm that people are noticing these internal bio markers, but provide the opportunity for them to discuss their day to day experiences of these lasting changes.

#### Recovery criteria

From a post-positivist perspective, there has been a longstanding call for standardized ED recovery criteria, typically involving weight, behavioral, and cognitive criteria (e.g., [14–17]). Drawing from the above, we suggest that research looking into recovery criteria may benefit from more diverse methodological approaches which pull from a variety of sources (e.g., clinicians, researchers, individuals with lived experience). For example, BMI has historically been used as an indicator of recovery status because it is readily obtained by ED researchers (and clinicians). While weight monitoring can be helpful in *specific* cases (e.g., those who are severely underweight or have lost a lot of weight in a short period of time), it is limited in use. Namely, BMI is insufficient to determine medical stabilization, it fails to take into account individual differences, and it can have negative impacts on treatment when individuals are discharged on the basis of weight alone [125]. Given these concerns, future research could discontinue the use of BMI as the “core” recovery criterion, as suggested by Kenny and Lewis [126], and instead focus on other variables that are more indicative of recovery over follow-up (e.g., Vall and Wade’s systematic review and meta-analysis findings of early symptom change during treatment as the most robust predictor of outcomes) [26].

Similarly, standardized assessments (e.g., EDE-Q, EDE, ED-LIFE) have been the “go-to” for assessing recovery outcomes in comparison to statistical norms. However, these measures are often developed by clinicians/researchers (thus reflecting what they feel is important in recovery) and in line with particular therapeutic modalities (e.g., the EDE-Q has a cognitive orientation). Thus, scores on these measures may not always match the person’s particular recovery aims and goals, nor the relative importance of particular behaviors in their lives. Employing these measures as a part of multi-methods designs with other types of assessments for comparison may offer the potential to think differently about these measures, and their role in assessing outcomes. We also suggest the need for measures co-designed with folks with lived experience and which reflect the diverse recovery elements described in qualitative studies (e.g., [33]).

#### The recovery process

Future research could compile more comprehensive lived experience narratives of changes in thought patterns through the recovery journeys (e.g., descriptions of how the “ED” voice began to leave, if ED voice is a relevant construct for the person), which could provide a more realistic timeline of this portion of the process for individuals and their “supports.” To begin to employ these kinds of measures in a way that opens up new possibilities, it would also be important to explore whether the ED-related ideas being measured resonate with the person whose recovery is being explored. Co-design processes may also be particularly relevant here, inviting people in recovery to be a part of research teams and take a role in determining the kinds of measures that could be used to assess recovery.

As years of ED behaviors and thoughts tend to impair different areas of psychosocial functioning (e.g., relationships, school/work, recreation, household duties), improvement in these areas, along with related QOL, tends to also lag behind physical recovery (e.g., [43]). Future research could further elaborate the timelines for which recovery in the different areas occurs, both from a group (e.g., through life story, narrative, or thematic analysis), and an individual (e.g., personal recording of recovery progress, case study approach) level. This approach offers a shift in methodological perspective, providing opportunity to conceptualize recovery differently.

#### Other areas for future consideration

Several studies (and informal support groups) have successfully employed recovered mentors, providing hope in recovery (e.g., [127]). Future research could examine more of the nuances of the mentorship role, including the characteristics of the mentor, the stage of recovery that the individual is in, and the dynamics of the mentor relationship. Further, taking a truly co-designed approach and, in particular, working with those who have not been included and heard in either treatment or research (not only more diverse participants, but their “supports,” including mentors), could offer new insight into recovery processes. Indeed, going forwards, we need to conduct our research differently if we want to incorporate the perspectives of those that we do not usually hear from.

Another area of study has developed around online (e.g., social media) use among those who are at risk for developing an ED, struggling with an ED, and those who are on the path to ED recovery. Analyses of online websites, blogs, and social media posts, along with their related potentially triggering content, have been conducted (e.g., [128]). However, on a positive note, this medium allows us to explore other methodological possibilities, including potentially focusing on reducing participant burden, and engaging with content from spaces where people are more “organically” describing these experiences, to get a sense of recovery outside of a clinical perspective. One possibility for future research is to combine the use of EMA with exposure to a range of different ED blog content (e.g., from triggering to supportive posts), in order to provide more proximal individual reaction information (e.g., EMA before exposure, EMA at exposure time, EMA after exposure time).

### Proposed future research model: Dialectical movement towards and away from a consensus

The aim of this paper is to offer a more diverse view of methodological perspectives (which stem from various ontological and epistemological stances) and ideas that might allow for forward movement in the field. As noted above, in a dialectal format, this can involve both movement toward *and* away from a consensus, including perspectives which do not seek to identify a single recovery definition. We believe that both can be simultaneously pursued, acknowledging that one does not discount the other. We have outlined several potential areas to explore which do not necessarily depend upon a consensus definition. Here, for balance, we would like to propose a future research model that could guide us in a direction that may eventually lead to a consensus definition–or definition*s*. In effect, we are advocating for: (1) transparency in researchers’ epistemological stances; (2) more varied approaches to research in order to “see” different aspects of recovery experiences; and (3) collaboration between researchers and other stakeholders to generate new methodological approaches and insights about recovery.

Our proposed future research model is detailed in Table 3. Based upon the studies we cited above, we suggest a multi-methods design (e.g., quantitative, qualitative, creative methods, psycho/physiological, behavioral, laboratory, online observations), which incorporates different perspectives (e.g., expanding recruitment of participants that have been less represented in the literature, including “supportive” others), and extends the duration of studies to provide a more longitudinal outlook (e.g., capturing cognitive recovery, and improvement in psychosocial functioning/QOL, which often comes later, and noting how definitions of recovery may change over time for people). In this way, we hope to move the field towards different, more nuanced, and comprehensive perspectives on ED recovery.

**Table 3:** Proposed potential future research model

Method types	Perspectives incorporated	Longitudinal design	Outcome measures
Quantitative	Individuals (increased diversity of races/ethnicities)	Multiple points of assessment for changes:	Risk Calculators (personalized risk of ED onset/relapse)
Qualitative	“Supports” (e.g., parents, family, partners, friends, mentors)	Symptoms	fMRI, DEXA scans (provide accurate body medical status)
Psycho/Physiological	Researchers	Cognitions	Time lines (when state and scar markers emerge)
Behavioral	Clinicians	Psychosocial functioning QOL	EMA “active” recovery (challenges, impacts on behavior/feelings)
Laboratory	Study co-designs (identify and assess aspects of ED recovery important to all constituents)	Recovery definitions	Participant-identified target behaviors (monitor decrease via standardized assessments for ongoing motivation)
Online Observations		Compare recovery operalizations vs. subsequent relapse rates	Participant narrative experiences of cognitive recovery (shifting identity from “ED” towards person)
Combine types for multi-methods design		Evaluation of BMI as predictive of recovery (vs. other variables)	Participant narrative experiences of psychosocial recovery (group: narrative thematic analysis of overall trends, individual: personal recording of progress)
		Provide group and individual level long term ED course trajectories	Supportive mentorship relationship aspects (mentor characteristics, stage of individual recovery, dynamics of the mentor relationship)
			Holding on to hope (more data indicates that recovery is possible, even after decades)

*BMI*  body mass index, *ED*  eating disorder, *EMA*  ecological momentary assessment, *DEXA*  dual energy X-ray Absorptiometry; fMRI = functional magnetic resonance imaging, *RC*  risk calculator, *QOL*  quality of life

## Conclusion

In conclusion, we would like to encourage a creative, transparent, and thoughtful approach to ED recovery methodology, that considers what each of the methods allows us to engage with, or not, as the case may be. What we can (and do) know about recovery is intricately tied to our methodological and study design choices, which all have limits. Within this context, while there is a benefit to current pushes in the field to “come to consensus,“ these consensus definitions *will necessarily* leave out some people and experiences. This is especially the case for those who have not been meaningfully included in the research we have conducted to reach this consensus (e.g., people with lived experience, “non-traditional” patients, patients without access to treatment). Since there are so many different facets of recovery experiences, using different methodologies is imperative to develop a more complete understanding.

Indeed, it is important to acknowledge how the centrality of the method that is chosen to define ED recovery in turn influences how researchers and clinicians understand recovery, and how one moves towards it. New insights into recovery processes may depend on new methods of investigation. Thus, we suggest that some potential areas for future research ideally employ multi-method designs (e.g., quantitative, qualitative, creative methods, psycho/physiological, behavioral, laboratory, online observations), incorporate different perspectives (e.g., expanding recruitment of participants that have been less represented in the literature, including supportive others) and extend the duration of studies to provide a more longitudinal outlook (e.g., capturing cognitive recovery, which often comes later, and noting how definitions of recovery may change over time for people). In this way, we hope to move the field towards different, more nuanced, and comprehensive perspectives on ED recovery.

## Data Availability

Not applicable.

## Abbreviations

AN: Anorexia nervosa
BED: Binge eating disorder
BMI: Body mass index
BN: Bulimia Nervosa
CHIME: Connectedness, hope and optimism, identity, meaning in life, empowerment
DEXA: Dual energy X-ray absorptiometry
EAT: Eating attitudes test
ED: Eating disorders
EDREQ: Eating Disorders Recovery Endorsement Questionnaire
EDRQ: Eating Disorders Recovery Questionnaire
EDQLS: Eating Disorders Quality of Life Scale
EDQOL: Eating Disorders Quality of Life
EEG: Electroencephalography
EMA: Ecological momentary assessment
ET: Eye tracking
fMRI: Functional magnetic resonance imaging
GEE: Generalized estimating equations
HeRQoLED: Health-Related Quality of Life in Eating Disorders Questionnaire
HLM: Hierarchical linear models
IPA: Interpretive phenomenological analysis
MANOVA: Multivariate analysis of variance
NIH: National Institutes of Health
RC: Risk calculator
SAMHSA: Substance Abuse and Mental Health Services Administration
SEAN: Severe and enduring anorexia nervosa
SEED: Severe and enduring eating disorder
QOL ED: Quality of life for eating disorders

## Footnotes

1 There are debates about the degree to which research can ever be truly objective or whether this is desirable. Here, we use objective and subjective in quotation marks to signal broader perceptions about these processes.
